# Magnetically Induced Current-Density Susceptibility of Circum[*n*]coronenes

**DOI:** 10.1021/acs.jpca.4c07293

**Published:** 2025-01-04

**Authors:** Qian Wang, Stefan Taubert, Dage Sundholm

**Affiliations:** Department of Chemistry, Faculty of Science, University of Helsinki, P.O. Box 55, A. I. Virtasen aukio 1, Helsinki FIN-00014, Finland

## Abstract

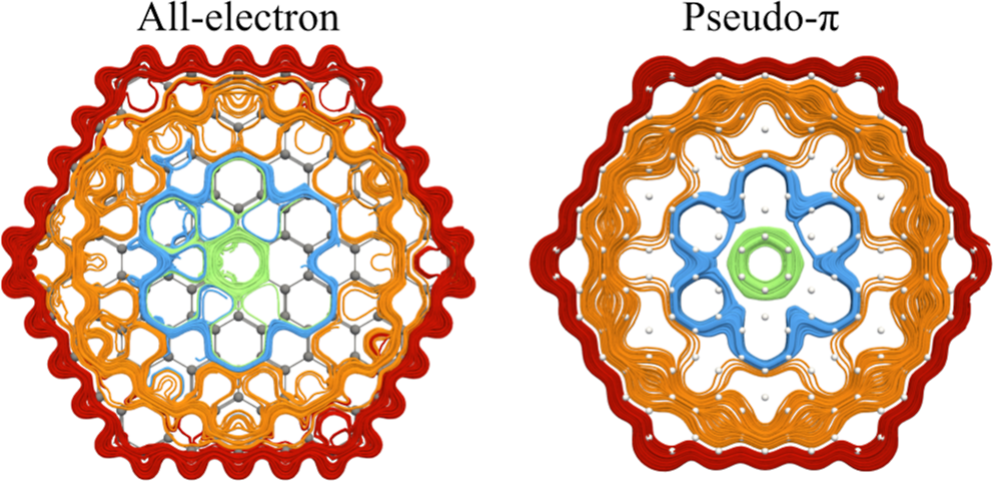

We have calculated the magnetically induced current density (MICD) susceptibility at the all-electron density functional theory level for a series of coronoid molecules of increasing size and compared the MICD susceptibilities with those calculated using the pseudo-π (PP) model. The molecules sustain global diatropic magnetically induced ring currents (MIRCs), whereas paratropic MICD vortices mainly appear inside the benzene rings. The computationally cheaper PP calculations were also employed on circum[*n*]coronene molecules showing that the MICD pattern continues to alternate for odd and even *n* when increasing the size of the molecule. For even *n*, there is a local paratropic MIRC in the middle of the molecule, whereas when *n* is odd, the PP models do not sustain any paratropic MIRC pathways. The global diatropic MIRC flowing mainly along the outer edge of the molecule increases with increasing *n* suggesting that there is no size limit of the MIRC of circum[*n*]coronene molecules. There are seven weakly aromatic Clar rings in the middle of the PP model of the circum[*n*]coronene molecules with odd *n*, whereas circum[*n*]coronene molecules with even *n* have no Clar rings. There are no Clar rings in the outer part of the circum[*n*]coronene molecules with *n* > 1.

## 1 Introduction

Coronene is the first member of the hexagonal polycyclic aromatic hydrocarbon (PAH) series. It has a diatropic magnetically induced ring current (MIRC) along the outer edge of the molecule, whereas the benzene ring in the middle sustains a paratropic MIRC.^1^ In hexabenzocoronene, which consists of coronene with six fused benzene rings at the outer edge, the benzene ring in the middle sustains a local diatropic MIRC as the six fused benzene rings also do. These seven benzene rings are locally aromatic Clar rings.^2−4^ Hexabenzocoronene also sustains a global diatropic MIRC along the outer edge.^5^ Circumcoronene consists of a coronene moiety in the center that is surrounded by 12 benzene rings; i.e., it has six benzene rings between the outer benzene rings of hexabenzocoronene. Circumcoronene, i.e., circum[*n*]coronene with *n* = 1, is the largest member of the coronoid series that has been synthesized. Telychko et al. synthesized circumcoronene on a Cu(111) surface.^6^ Zou et al. were very recently able to synthesize circumcoronene in solution.^7^ Based on calculations of its magnetic properties, Zou et al.^7^ concluded that it is a Clar aromatic molecule sustaining local MIRC in the seven benzene rings of the ideal Clar structure.^3,4^

The magnetic properties of circumcoronene have also previously been studied computationally by many other research groups.^1,2,8−11^ Circumcoronene also sustains a global MIRC along the outer edge of the molecule. Steiner et al. showed that the approximate pseudo-π (PP) method provides qualitatively the same magnetically induced current density (MICD) as obtained in all-electron calculations.^8,12^ Thus, the MICD of very large circum[*n*]coronene (*n* ≫ 1) molecules can be investigated by using the computationally cheaper PP method. Zou et al.^7^ used the anisotropy of the induced current density (AICD) approach^13,14^ showing that circumcoronene sustains local MIRCs in the seven Clar rings. However, the strength of the MIRC in the Clar rings cannot be quantitatively determined using AICD calculations because the scalar AICD function does not fulfill the charge-conservation condition.^15^ Therefore, the conclusions drawn from AICD calculations depend on the threshold used with the AICD function.

In this work, we computationally study pathways of the MICD of circum[*n*]coronene molecules by calculating profiles of the MICD strength. The main MICD profile yielding the strength of the MIRC around the molecular center is obtained by integrating the MICD passing through a plane that radially cuts the molecule from the geometrical center to a very large distance outside the outer edge of the molecule, where the MICD vanishes. The strengths of other MICD pathways are obtained by calculating the profile of the MICD passing through a number of planes that are parallel to the main plane and cut chemical bonds in the circum[*n*]coronene molecules.

In the next section, we describe the employed computational methods. In the first part of Section 3, we discuss the MICD susceptibility obtained at the all-electron level for the five smallest molecules. The MICD values calculated at the PP level for the same molecules as well as for larger circum[*n*]coronene molecules are discussed in the second part of Section 3. The main results are summarized in Section 4.

## 2 Computational Methods

The electronic structure of benzene, coronene, and circum[*n*]coronene (*n* = 1, 2, and 3) (denoted AE1–AE5) was calculated at the density functional theory (DFT) level using Turbomole.^16−18^ The molecular structures were fully optimized at the DFT level using the CAM-B3LYP^19^ functional and the def2-SVP basis sets.^20^ The Cartesian coordinates are reported in the Supporting Information. The molecules were assumed to belong to the *D*_6h_ point group. The nuclear magnetic resonance (NMR) shielding tensors of AE1–AE5 were calculated using gauge-including atomic orbitals^21,22^ at the CAM-B3LYP level with the mpshift program of Turbomole.^23,24^ Gauge-independent MICD susceptibilities were calculated with the GIMIC program using the one-electron density matrix and magnetically perturbed density matrices in the atomic orbital basis. The density matrices were obtained in DFT and nuclear magnetic shielding calculations. The Cartesian coordinates of the molecular structures and the basis-set information are also input data to the GIMIC program.^1,5,25−27^ The MICD is visualized using Paraview.^28^ The D1 diagnostics was calculated for AE4 and AE5 at the second-order Møller–Plesset (MP2/def2-SVP) perturbation level.^29−31^ The lowest singlet and triplet excitation energies were also calculated for AE4 and AE5 at the approximate second-order coupled-cluster (CC2/def2-SVP) level using the reduced-virtual-space approach.^31−33^

The Cartesian coordinates of the molecular structure of the molecules studied at the PP level (denoted PP1–PP20) were obtained using a freely available python script.^34^ The MICD susceptibilities of PP1–PP10 were calculated at the PP level^12^ using the def2-TZVP basis sets.^35^ For PP11–PP20, we used the def2-SVP basis sets in the PP calculations.^20^ The PP calculations were performed at the HF level as well as at the DFT level using the CAM-B3LYP^19^ and the ωB97X-D^36^ functionals because the functional must have 100% HF exchange at long electron–electron distances to avoid spurious charge transfer in the largest molecules. The NMR shielding tensors and nucleus independent chemical shifts (NICSs)^37^ were calculated at the same levels of theory with the mpshift module of the Turbomole program. The results obtained in the PP11–PP20 calculations are reported only in the Supporting Information. The results obtained for these molecules are less reliable because the calculations suffer from computational difficulties that we discuss in the next section. NICS values are reported only in the Supporting Information.

Since GIAOs were used in the calculations of the NMR shielding tensors, the calculated MICD susceptibility has no reference to any gauge origin. The MIRC was obtained by contracting the MICD susceptibility with an external magnetic field applied perpendicularly to the molecular plane.^1,5,25−27^

**Figure 1:**
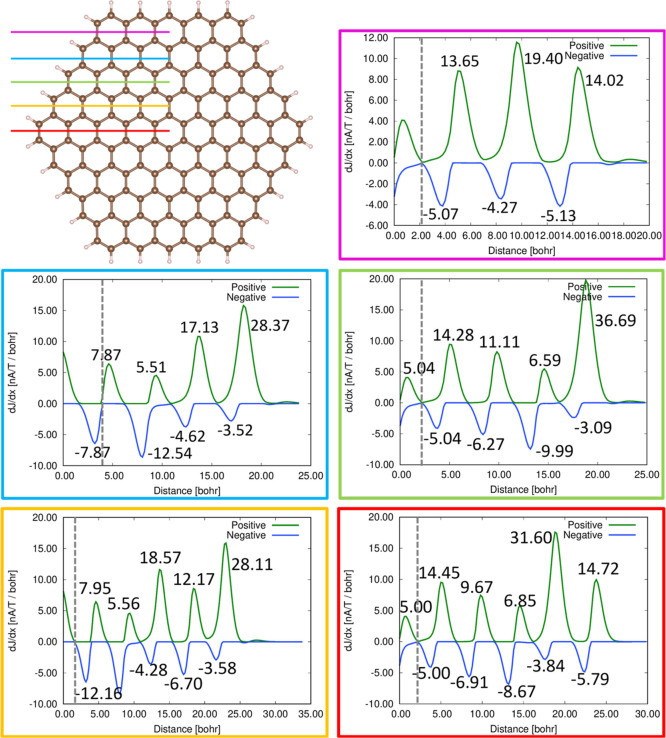
Integration planes for determining the strength of the MIRC pathways along different chemical bonds of circum[3]coronene (AE5). The frame of the MIRC profile pictures has the same color as the integration plane. The contributions to the MIRC strength from different parts of the molecule are given. The MIRC profiles of AE1–AE4 and PP1–PP10 are reported in the Supporting Information. The integration begins in the middle of the molecule at the dashed line. The molecular structure has been made with Vesta^40^ and the profile pictures with Gnuplot.^41^

The aromatic nature is determined from the direction of MIRC and its strength. Other aromaticity criteria have also recently been discussed.^38^ Molecules sustaining a net diatropic (flowing in the classical direction) MIRC are aromatic, whereas in antiaromatic molecules, the MIRC flows in the paratropic (in the opposite) direction.^1,5,25,26^ The strength of the MIRC is obtained by integrating the MICD passing through a plane that begins in the center of the molecule and extends far away from the molecule, where the MICD vanishes. The integration plane coincides with one of the σ_v_ planes of the *D*_6h_ point group and passes through the middle of the C–C bond at the corner of the molecule. We determine the strength of the MIRC pathways by calculating the profiles of the MICD passing through several integration planes and integrating the diatropic and paratropic contributions separately.^1,5,25−27,39^ The integration planes are shown in Figure 1.

The unit of the MIRC (susceptibility) strength is nAT^–1^. The MIRC strengths can also be calculated by line integration of the σ_*zz*_ component of the magnetic shielding tensor in the interval ,^42^ where *z* coincides with the *C*_6_ axis of the *D*_6h_ point group. The *z* axis is also the direction of the external magnetic field. The diatropic and paratropic contributions to the MICD susceptibility can be identified and visualized separately.^39,43^

## 3 Results and Discussion

### 3.1 All-Electron Calculations

The energy gap between the highest occupied molecular orbital (HOMO) and the lowest unoccupied molecular orbital (LUMO) obtained in the all-electron (AE) calculations decreases with increasing size of the molecule, as seen in Table 1. The table also shows that the average ^1^H NMR shielding constant decreases with an increasing size of the molecule because the strength of the MIRC increases. Integration of the MICD yielded a net MIRC of 12.1 nAT^–1^ for benzene compared to the MIRC strength of 47.1 nAT^–1^ for circum[3]coronene. The diatropic and paratropic contributions to the strength of the MIRC as well as the net MIRC strengths are reported in Table 1. Thus, the larger circum[*n*]coronenes are more aromatic than the smaller ones according to the ring-current criterion. The ^1^H NMR signals of the large circum[*n*]coronene molecules are shifted to larger chemical shifts (smaller magnetic shielding values) since they sustain a strong diatropic MIRC.

**Table 1:** HOMO–LUMO Gap (in eV), the Average ^1^H NMR Shielding Constant ( in ppm), the Strength of the Diatropic MIRC (Dia in nAT^–1^), the Strength of the Paratropic MIRC (Para in nAT^–1^), and the Net MIRC (Net in nAT^–1^) of Benzene, Coronene, and Circum[*n*]coronene with *n* = 1, 2, and 3

molecule	gap	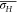	dia	para	net
benzene	9.55	24.18	17.3	–5.2	12.1
coronene	6.24	22.50	27.2	–16.4	10.7
circum[1]	4.67	20.87	45.7	–16.8	29.0
circum[2]	3.72	19.50	55.3	–28.5	26.8
circum[3]	3.05	18.31	77.3	–30.2	47.1

The diatropic and paratropic contributions to the net MIRC strengths reported in Table 1 show that circum[2]coronene with even *n* has a weaker MIRC than circumcoronene because the innermost benzene ring of circum[2]coronene sustains a paratropic MIRC, whereas circumcoronene (*n* = 1) sustains a diatropic MIRC around the innermost benzene ring. The alternating pattern continues for the larger circum[*n*]coronene molecules.

The alteration of the MICD character can be understood by counting the number of π electrons in the conjugated bonds. The number of carbon atoms, i.e., the number of π electrons in the conjugated bonds of the circum[*n*]coronene molecules, is 6(*n* + 2)^2^. Every second molecule in the circum[*n*]coronene series has then (4*N* + 2) π electrons (54, 150, ... for *n* = 1, 3, ...) in the conjugated π orbitals and every second molecule has 4*N* (24, 96, ... for *n* = 0, 2, ...) electrons in the conjugated π orbitals. Assuming that the hexagonal circum[*n*]coronene molecules consist of a circular disc with a uniform π-electron density, the molecules with (4*N* + 2) π electrons are globally aromatic because the aromaticity rules of a circular disc are the same as for a ring.^44−46^ Every second molecule in the series has 4*N* π electrons in the conjugated bonds, leading to antiaromaticity. However, the whole molecule does not become antiaromatic sustaining a global paratropic MIRC. Instead, the conjugated orbitals in the innermost benzene ring sustains a local paratropic MIRC. The rest of the conjugated orbitals contain then (4*N* + 2) π electrons leading to aromaticity, i.e., they sustain a global diatropic MIRC.

**Figure 2:**
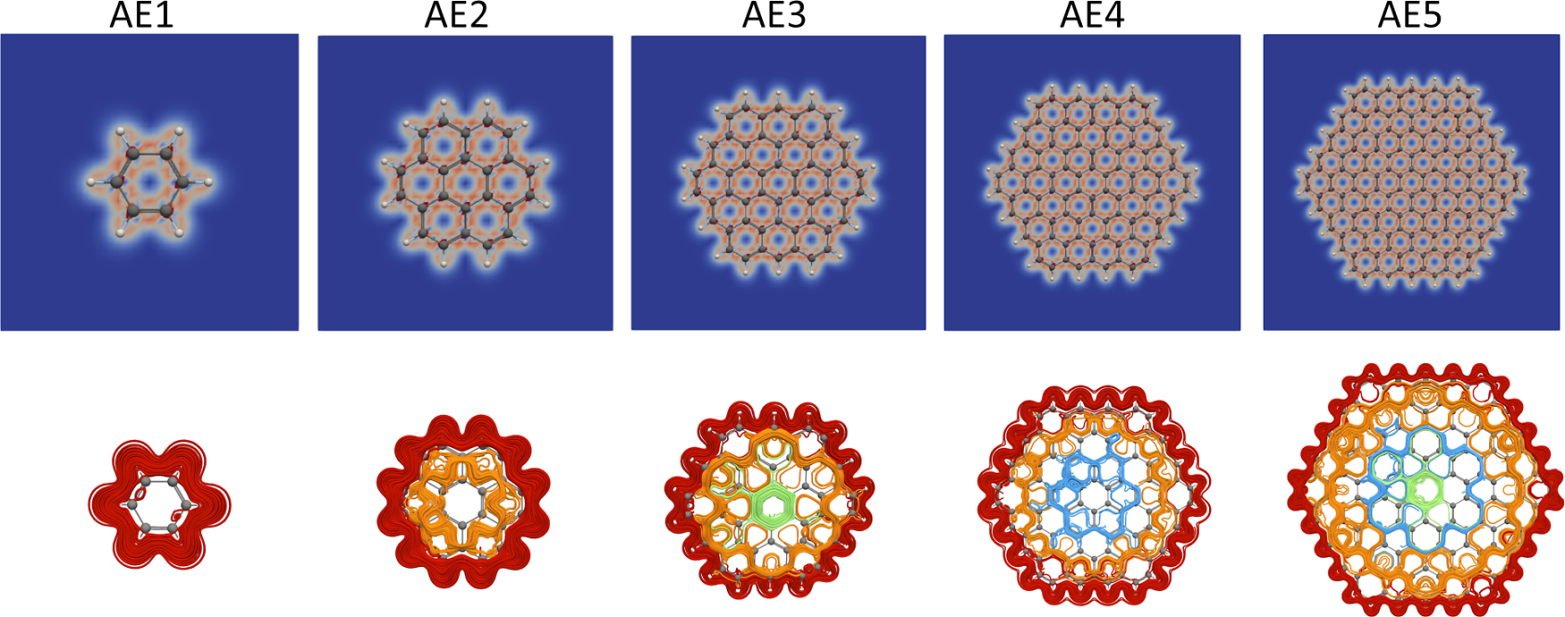
MICD susceptibility obtained in the AE calculations on benzene, coronene, and circum[*n*]coronene (*n* = 1, 2, and 3) is shown in the upper pictures. The strongest MICD is indicated with red, weaker MICD is white, and blue means no MICD. The main MIRC pathways are shown in the lower picture. Different colors are used to show the main MIRC pathways. The figure has been made with Paraview.^28^

The MIRC pathways of benzene, coronene, and circum[*n*]coronene (*n* = 1, 2, and 3) are shown in Figure 2. The strongest MIRC pathways follow along the outer edge of the molecules or near it. The MIRC pathways in the interior of the molecules are more complicated and weaker. There is a coronene-shaped MICD in the middle of the circum[*n*]coronene molecules with even *n* but not in the ones with odd *n*. The MIRC along the edges of the molecules splits at the corners. For circum[2]coronene, the strengths of the two branches are about the same, whereas for circum[3]coronene, the inner branch is 10 nAT^–1^ stronger than the outer one.

**Figure 3:**
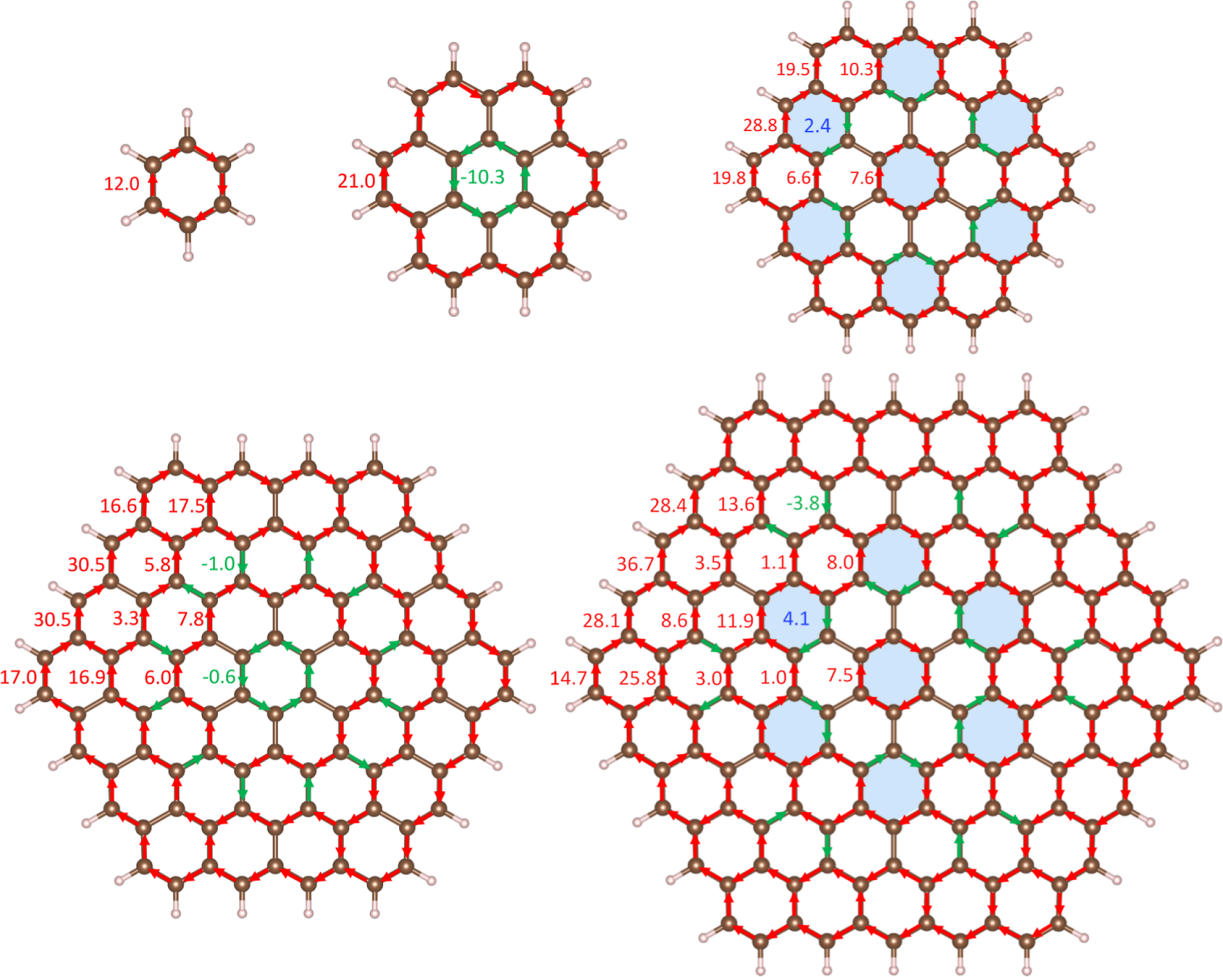
MIRC pathways in benzene, coronene, and circum[*n*]coronene (*n* = 1, 2, and 3). The strength of the diatropic MIRC along chemical bonds is given in red and the strengths of the MIRC flowing locally in the opposite direction are given in green. Blue areas indicate Clar rings sustaining a net local diatropic MIRC. The molecular structure has been made with Vesta.^40^

The integration planes for circum[3]coronene shown in Figure 1 were used for determining the strength of the MIRC passing along the chemical bonds. The strengths were obtained by integrating the MICD contributions passing through the planes. The planes begin at the *C*_2_ symmetry axis and extend to a large distance outside the molecule, where the MICD vanishes. The strengths of the MIRC passing chemical bonds are reported in Figure 3, where the Clar rings sustaining a weak local diatropic MIRC are colored blue.

Circumcoronene has seven Clar rings.^7^ They sustain a weak MIRC of 2.4 nAT^–1^, which is only 20% of that for benzene. Molecules, whose ring current is weaker than ±3 nAT^–1^, can be considered nonaromatic.^47^ The Clar ring in the middle sustains a diatropic MIRC of 7.6 nAT^–1^. However, there is a paratropic MIRC of −5.1 nAT^–1^ inside the Clar ring yielding a net MIRC of only 2.5 nAT^–1^. Circum[2]coronene has no Clar rings. Circum[3]coronene has seven Clar rings in the middle of the molecule. Six of them sustain a MIRC of 4.1 nAT^–1^, whereas the strength of the MIRC of the Clar ring in the middle is only 2.5 nAT^–1^ because there is a paratropic MIRC of −5.0 nAT^–1^ inside the ring. The alternating MICD character continues for the larger circum[*n*]coronene molecules, according to the PP calculations. The obtained Clar structures of the circum[*n*]coronenes with *n* > 1 do not agree with the previously reported ones.^48^

Circum[*n*]coronenes larger than circumcoronene have previously been predicted to have significant open-shell multiradical character.^48−50^ Even though the HOMO–LUMO gap of circum[3]coronene is 3.05 eV, we found that the lowest singlet and triplet states at the DFT level using the CAM-B3LYP functional are almost degenerate. Our calculations at the CAM-B3LYP level suggest that circum[*n*]coronenes with *n* > 3 have a significant multiradical electronic structure. Circum[3]coronene is the borderline case. However, MP2 calculations on circum[2]coronene and circum[3]coronene did not indicate any multiradical character, because the D1 diagnostic values are 0.0347 and 0.0405, respectively. The lowest singlet and triplet excitation energies (in eV) of circum[2]coronene calculated at the CC2 level for each irreducible representation of the *D*_2h_ point group are 2.58 (^1^A_*g*_), 2.67 (^1^B_1*g*_), 5.11 (^1^B_2*g*_), 5.11 (^1^B_3*g*_), 5.61 (^1^A_*u*_), 5.61 (^1^B_1*u*_), 2.12 (^1^B_2*u*_), 1.83 (^1^B_3*u*_), 2.19 (^3^A_*g*_), 2.19 (^3^B_1*g*_), 5.09 (^3^B_2*g*_), 5.09 (^3^B_3*g*_), 5.58 (^3^A_*u*_), 5.58 (^3^B_1*u*_), 1.61 (^3^B_2*u*_), and 1.84 (^3^B_3*u*_). The corresponding excitation energies (in eV) of circum[3]coronene are 2.03 (^1^A_g_), 2.03 (^1^B_1g_), 5.10 (^1^B_2g_), 5.10 (^1^B_3g_), 4.77 (^1^A_u_), 4.76 (^1^B_1u_), 1.67 (^1^B_2u_), 1.43 (^1^B_3u_), 1.62 (^3^A_g_), 1.62 (^3^B_1g_), 5.08 (^3^B_2g_), 5.08 (^3^B_3g_), 4.76 (^3^A_u_), 4.76 (^3^B_1u_), 1.21 (^3^B_2u_), and 1.42 (^3^B_3u_). All excitation energies are larger than 1 eV and the D1 diagnostic values are small, suggesting that AE4 and AE5 have closed-shell wave functions dominated by a single Slater determinant.

The total paratropic contribution to the MIRC of coronene is −16.4 nAT^–1^ and the diatropic contribution is 27.2 nAT^–1^. These MIRC strengths also contain contributions from local paratropic MIRC inside the outer benzene ring. The net MIRC of 10.7 nAT^–1^ consists of a paratropic MIRC of −10.3 nAT^–1^ around the benzene ring in the middle and a diatropic MIRC of 21.0 nAT^–1^ along the edge.

The net MIRC of the entire circumcoronene molecule is 29.0 nAT^–1^. It consists of a diatropic contribution of 47.7 nAT^–1^ and a paratropic contribution of −16.8 nAT^–1^. The benzene ring in the middle of circumcoronene sustains a local diatropic MIRC of 7.6 nAT^–1^. The MIRC of 26.4 nAT^–1^ along the edge splits into the corners of the molecule. The strength of the inner branch is 6.6 nAT^–1^ and 19.8 nAT^–1^ along the outer pathway. The weak diatropic local MIRC of 2.4 nAT^–1^ in the Clar ring at the edge between the corners leads to a MICD strength of 28.8 nAT^–1^ in the middle of the edges. The two global MIRC pathways are colored red and orange in Figure 2.

The circum[2]coronene molecule sustains a net MIRC of 26.8 nAT^–1^, which is slightly smaller than that for circumcoronene because of the strong paratropic ring current of −12.7 nAT^–1^ inside the benzene ring in the middle of the molecule, whereas there is practically no diatropic MIRC around the innermost benzene ring. The coronene moiety in the center sustains a net diatropic MIRC of 6.0 nAT^–1^. The MIRC along the outer part of circum[2]coronene splits at the rings in the corners into two almost equally strong pathways of 17 nAT^–1^. The two branches join leading to a MIRC strength of 30.5 nAT^–1^ along the edge and only 3.3 nAT^–1^ continues along the inner pathway. The two pathways are colored red and orange in Figure 2. Inspecting MIRC pathways and their strengths shows that there are no Clar rings in circum[2]coronene.

The main MIRC pathways of circum[3]coronene also consist of a MIRC along the edge that splits into an inner and an outer branch in the corners, as shown in red and orange in Figure 2. The inner pathway of 25.8 nAT^–1^ is stronger at the benzene ring in the corner, whereas the main MIRC passes along the edge between the corners as shown in Figure 3. The MICD of the inner part of circum[3]coronene with seven local weakly aromatic Clar rings is reminiscent of the MICD in the middle of circumcoronene. The strengths of the MIRC passing different bonds are shown in Figure 3.

Separation of the MICD into diatropic and paratropic contributions shows that the paratropic contributions appear inside the benzene rings.^39^ It is stronger inside the benzene ring in the middle of coronene and circum[2]coronene. The separated diatropic and paratropic MICD are shown in the Supporting Information.

### 3.2 PP Calculations

The molecular structures used in the PP calculations (PP1–PP20) were generated by using a Python script^34^ without further optimization. The reliability of the PP approach was assessed by performing PP calculations on the smaller molecules for which AE results are available. In the PP approach, the hydrogen atoms along the edge are removed and the carbon atoms are replaced by hydrogen atoms.^12^ The idea of the PP approach is that there is a one-to-one correspondence between the σ orbitals of the PP models and the π orbitals of the AE calculations. The PP model is a topological model that is expected to provide the same aromatic nature as that obtained in the AE calculations. The computational costs of the PP calculations are small because the number of electrons is about one-sixth of the one in the AE calculations on the coronoid molecules and the number of basis functions is much smaller than at the AE level because the studied system consists of only hydrogen atoms. In addition, a small basis set is usually enough for a qualitative description of MICD at the PP level. However, since the calculations are fast, we used standard basis sets to avoid uncertainties originating from the basis-set incompleteness.

MICD calculations were performed on the PP1–PP10 models at the HF and DFT levels by using the CAM-B3LYP functional. The MICD calculations for the PP11–PP20 models reported in the Supporting Information were performed at the DFT level using the ωB97X-D functional. The diatropic and paratropic contributions to the MICD of the PP1–PP10 models are shown in Figure 5, whereas the ones for PP11 to PP20 are shown in the Supporting Information. The MICD calculated for PP1–PP5 is very similar to the ones obtained for benzene, coronene, and circum[*n*]coronene molecules with *n* = 1, 2, and 3.

**Figure 4:**
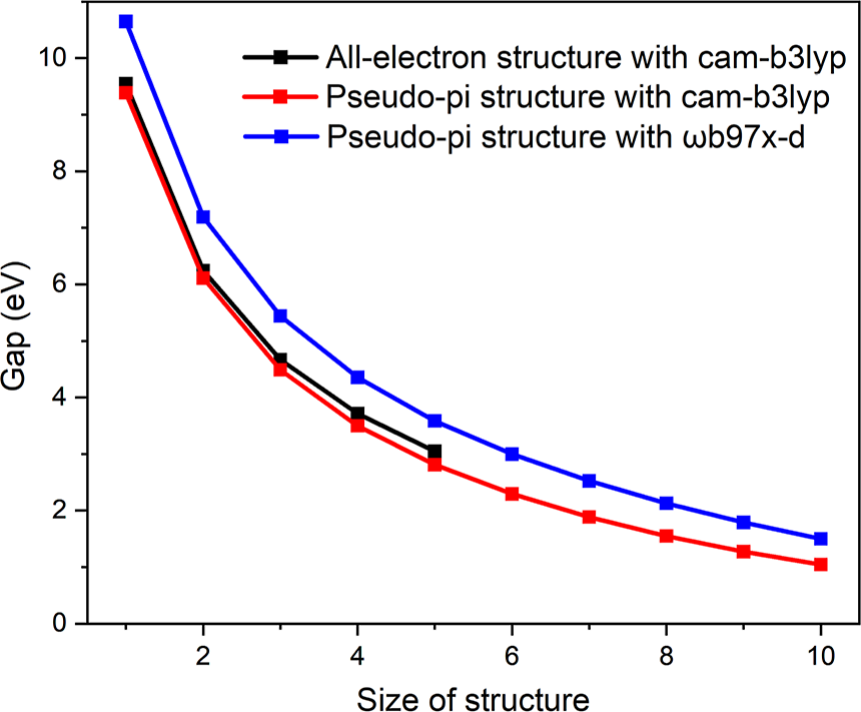
HOMO–LUMO gap calculated for the studied molecules at different levels of theory. The figure has been made with Origin Pro.^51^

The PP calculations on the PP1–PP5 models yielded practically the same HOMO–LUMO gap as obtained in the corresponding AE calculations. The gaps calculated at the AE and PP levels are compared in Figure 4 and in Tables 1 and 2, where one sees that the gap decreases in the same way at the employed levels of theory with increasing size of the molecule. Since the HOMO–LUMO gaps calculated at the two levels for the smaller molecules agree well, we also calculated them for the larger PP models even though their ground state is not a closed-shell singlet state. Their ground state is a high-spin state or an open-shell singlet state, whose wave function is not well described by one Slater determinant. The HOMO–LUMO gaps calculated at the ωB97X-D level are slightly larger than the ones obtained in the CAM-B3LYP calculations, probably due to the larger amount of Hartree–Fock (HF) exchange when the electrons are far apart. For very large PP models, a large amount of long-ranged HF exchange is necessary for preventing spurious charge separation that causes problems when calculating the MICD. Irregularities in the magnetic properties begin to appear for PP11 at the ωB97X-D and CAM-B3LYP levels, and they show up for PP14 at the HF level. DFT calculations on PP6 and larger PP models suggest that their ground state is a symmetry-broken high-spin state, which is an artifact of the employed computational levels. Even though we report MICD calculations for PP11 to PP20 in the Supporting Information, the MICDs must be considered with care due to the limitations of the employed computational levels to describe the electronic structure of large circum[*n*]coronene molecules. A spatial multiconfigurational character of the wave function is expected to appear along the edge and in the outer part of the molecules, where the length of the C–C bonds alternate for the large molecules, whereas in the middle, the C–C bonds must be more or less equal due to the confinement.

**Table 2:** HOMO–LUMO Gap (in eV), the Strength of the Diatropic MIRC (Dia in nAT^–1^), the Strength of the Paratropic MIRC (Para in nAT^–1^), and the Net MIRC Strength (Net in nAT^–1^) of the PP1–PP10 Models Calculated at the PP Level Using the CAM-B3LYP Functional

system	gap	dia	para	net
PP1	9.39	12.4	0	12.4
PP2	6.11	17.9	–5.8	12.1
PP3	4.49	32.2	–0.2	31.9
PP4	3.50	38.9	–7.5	31.9
PP5	2.81	55.1	–1.9	53.1
PP6	2.29	66.3	–6.5	59.8
PP7	1.88	82.2	–1.9	80.3
PP8	1.55	89.9	–6.5	83.4
PP9	1.27	104.1	–1.9	102.2
PP10	1.05	110.1	–5.0	105.1

The same alternating nature of the MICD is seen in the PP1–PP5 models as that at the AE level. The pattern continues in the PP models until PP12. For PP models larger than PP12, the alternation continues, but there is a local paratropic MIRC inside many benzene rings in the interior of the molecule and local paratropic contributions to the MICD also appear at the edges. The change in the nature of the MICD suggests that the calculations on the large PP models are less reliable. The PP*n* models with even *n* sustain local paratropic MIRC in the seven Clar rings in the middle, whereas the PP*n* models with odd *n* sustain only diatropic contributions to the MICD. Since the PP calculations simulate the MICD contributions of the π orbitals, there are generally no local paratropic MIRC inside the rings at the PP level because the local paratropic MIRC mainly originates from electrons in the σ orbitals.

**Figure 5:**
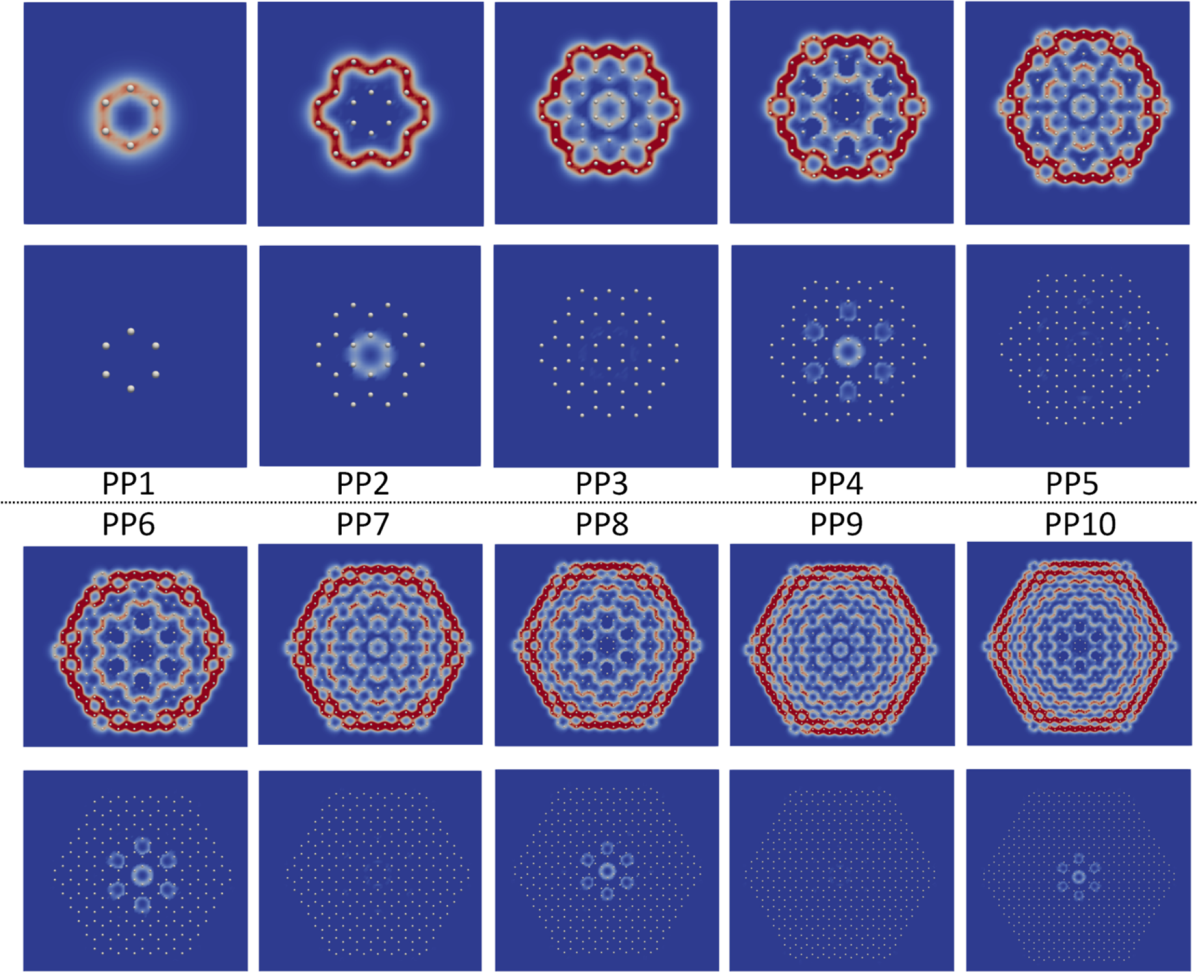
Diatropic (upper pictures) and paratropic (below) contributions to the MICD of the PP models calculated at the CAM-B3LYP level. The relative MICD strength is shown with colors, where red means the strongest and then white. Blue means no current density. The figure has been made with Paraview.^28^

The PP6–PP10 models sustain an MICD pathway along the edge that splits at the corners. The MICD on the outside of the benzene rings in the corners is weak for the larger PP models, whereas the MIRC splits at the benzene rings on both sides of the benzene rings in the corners. For the PP8–PP10 models, the MICD is slightly shifted inward in the corners. The MICD of PP4 and PP5 is compared to the ones calculated at the AE level in the Supporting Information. Figure 5 shows that the larger PP*n* models do not exhibit any diatropic Clar rings outside the central part. The ones with even *n* have seven paratropic Clar rings in the middle of the molecule. Large PP*n* models with odd *n* do not sustain any paratropic MICD, whereas they have seven diatropic Clar rings in the middle of the molecule.

## 4 Summary and Conclusions

The MICD susceptibility has been calculated at the AE DFT level for benzene, coronene, and circum[*n*]coronene, where *n* = 1, 2, and 3. The MICD has also been calculated for them at the PP level. Qualitatively, the same MICD is obtained in the AE and PP calculations. There is a strong MIRC pathway along the outer edge, which splits at the benzene rings in the corners of the circum[*n*]coronene molecules.

The MICD character alternates with *n*. The alternating nature of the MICD can be understood by assuming that the hexagonal molecules are a circular disc, and each carbon atom contributes one electron to the conjugated bonds. Circum[*n*]coronene molecules with odd *n* are then aromatic with 4*N* + 2 electrons in the conjugated bonds, whereas those with even *n* fulfill the aromaticity rule for antiaromatic molecules that sustain a paratropic local ring current around the benzene ring in the middle. The rest of the molecule has 4*N* + 2 electrons in the conjugated bonds, sustaining a diatropic MIRC.

The circum[*n*]coronene molecules with even *n* have seven Clar rings sustaining a weak local diatropic MIRC of about 2–4 nAT^–1^. A similar MICD pattern with seven Clar rings in the middle was previously obtained for another series of hexagonal PAHs with 4*N* + 2 electrons in the conjugated bonds.^8^ Hexabenzocoronene with 4*N* + 2 electrons in the conjugated bonds also fulfills the aromaticity rule for a round aromatic disc and has seven Clar rings.^1^ There are no Clar rings in the circum[*n*]coronene molecules with even *n*. The ground state of the smaller molecules is a closed-shell singlet state, whereas the lowest singlet and triplet states are almost degenerate for circum[3]coronene, which is the largest molecule that was studied at the AE level. The ground state of the larger circum[*n*]coronene molecules has open-shell character.

The MICD calculated at the PP level for circum[*n*]coronene with *n* = 4–8 shows that the alternating MICD character continues. We report the MICD for circum[*n*]coronene with *n* = 9–18 only in the Supporting Information because they are less reliable. The MICD character becomes irregular when *n* > 10 suggesting that spurious charge transfer appears in addition to the significant open-shell character of the wave function.

The strength of the MIRC increases with increasing size of the circum[*n*]coronene molecules suggesting that there is no size limit for their diatropic MIRC, which has been previously suggested for other kind of molecular rings.^52^

## Supporting Information Available

Pictures of the MICD susceptibilities, current-density strengths, current-density profiles, and ^1^H NMR magnetic shielding constants (PDF)
Cartesian coordinates of the optimized molecular structures (ZIP)
