# Supplementary material for: Magnetically Induced Current-Density Susceptibility of Circum[n]coronenes
Source: J Phys Chem A. 2025 Jan 4;129(2):527–35. doi: 10.1021/acs.jpca.4c07293 (PMC11744783; doi:10.1021/acs.jpca.4c07293)
Supplement: Supplementary file 1 — jp4c07293_si_001.pdf [file jp4c07293_si_001.pdf]

Magnetically Induced Current-Density Susceptibility of Circum[n]coronenes

Supporting Information

Qian Wang, Stefan Taubert, and Dage Sundholm*

Department of Chemistry, Faculty of Science, University of Helsinki, P.O. Box 55, A. I. Virtasen aukio 1, FIN-00014 Helsinki, Finland

E-mail: dage.sundholm@helsinki.fi

Circum[n]coronenes studied at the all-electron (AE) level

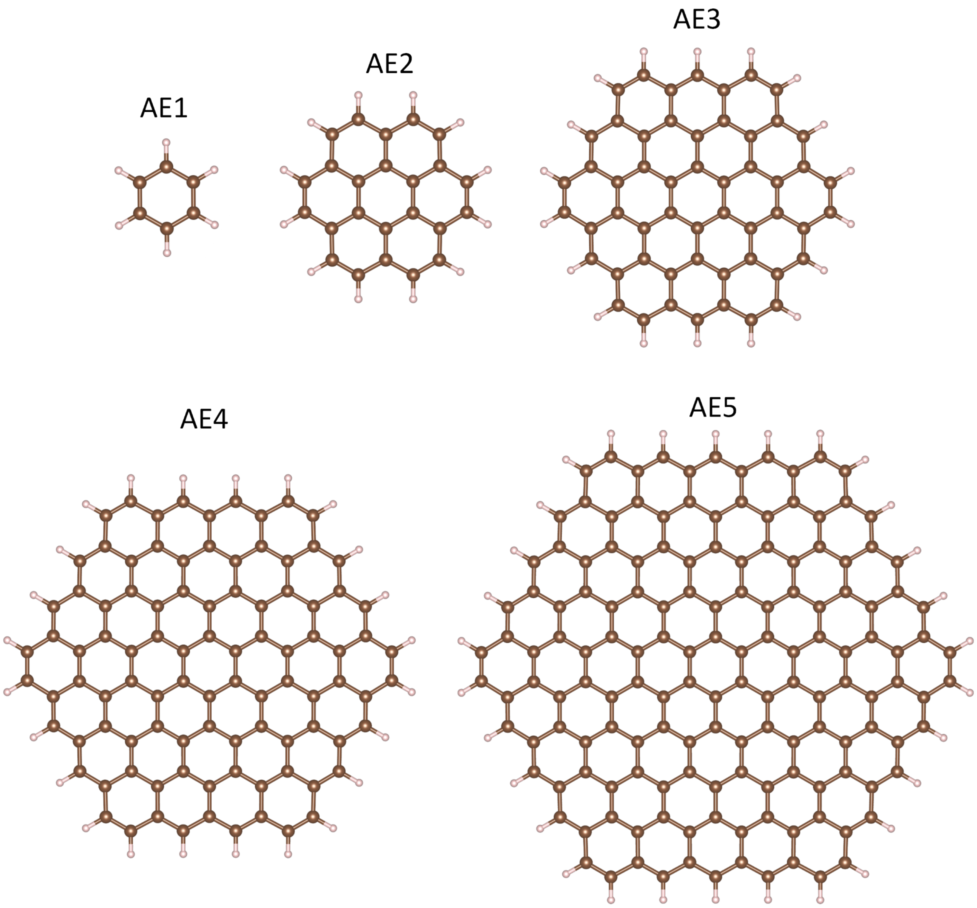

Figure S1: The molecular structure of benzene, coronene, and circum[n]coronene with $n = 1, 2, 3$. Carbon atoms are brown and the hydrogen atoms are white. The pictures have been made with Vesta.[1]

Pseudo-π models of the circum[n]coronenes

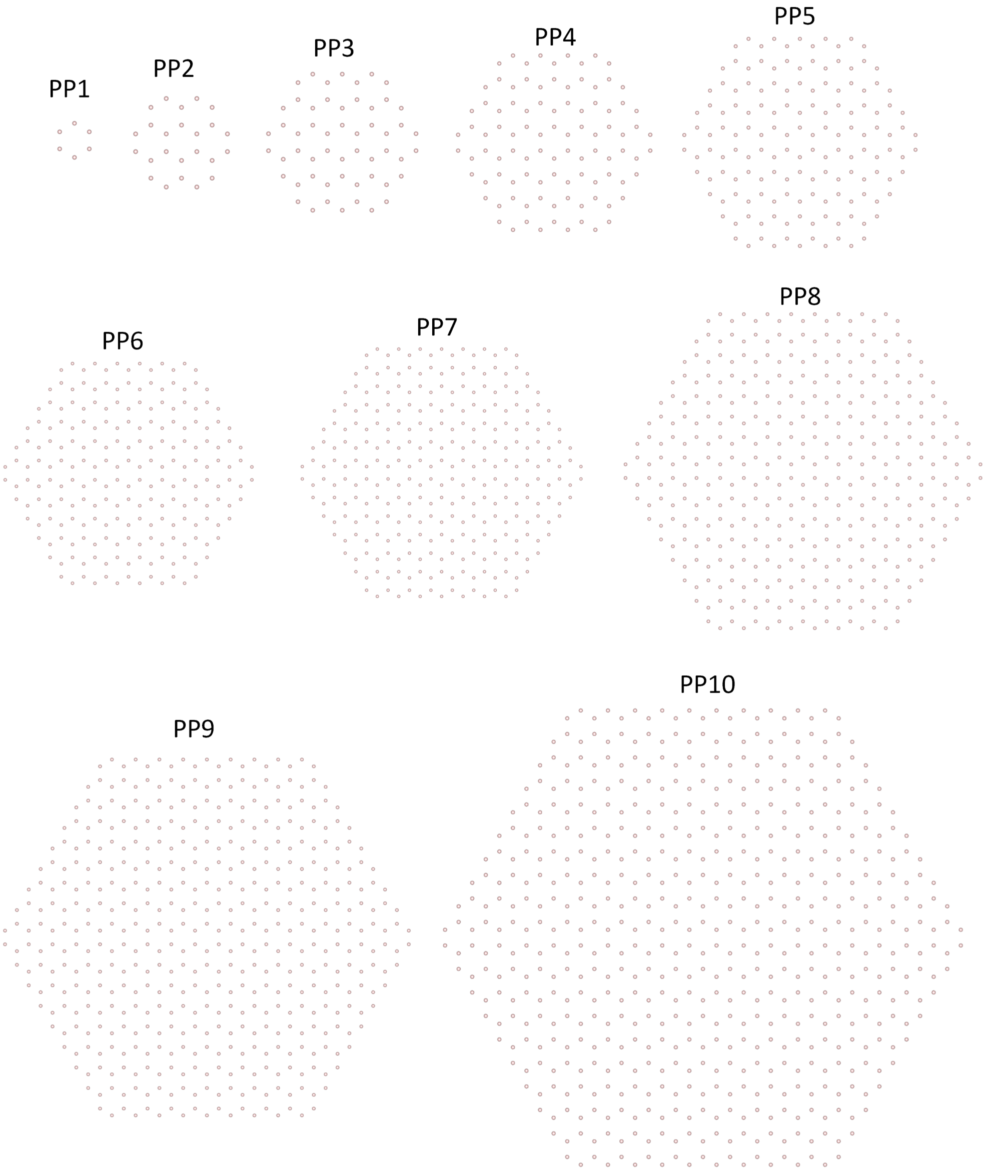

Figure S2: The molecular structures of the 10 smallest pseudo-π models of the circum[n]coronene molecules. All atoms are hydrogen. The pictures have been made with Vesta.[1]

The current density studied at the AE level

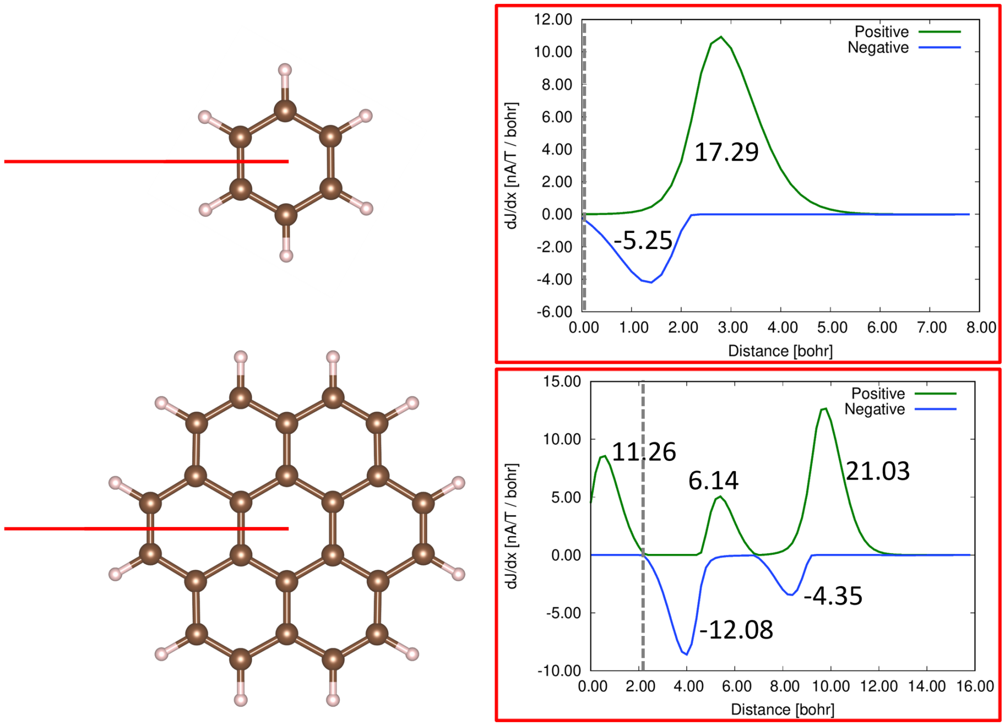

Figure S3: The integration plane and the profile of the magnetically induced ring current (MIRC) of benzene (AE1) and coronene (AE2). The integration begins in the middle at dashed line. The molecular structures have been made with Vesta[1] and the profile pictures with Gnuplot.[2]

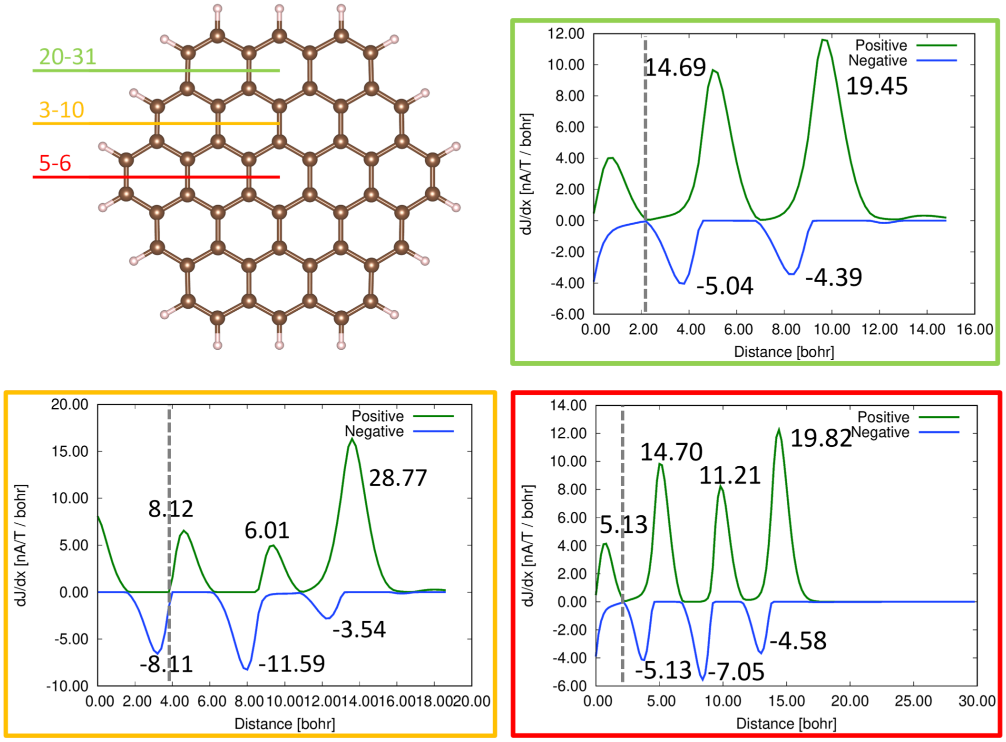

Figure S4: The integration plane and the MIRC profile of circumcoronene (AE3). The integration begins in the middle at dashed line. The molecular structures have been made with Vesta[1] and the profile pictures with Gnuplot.[2]

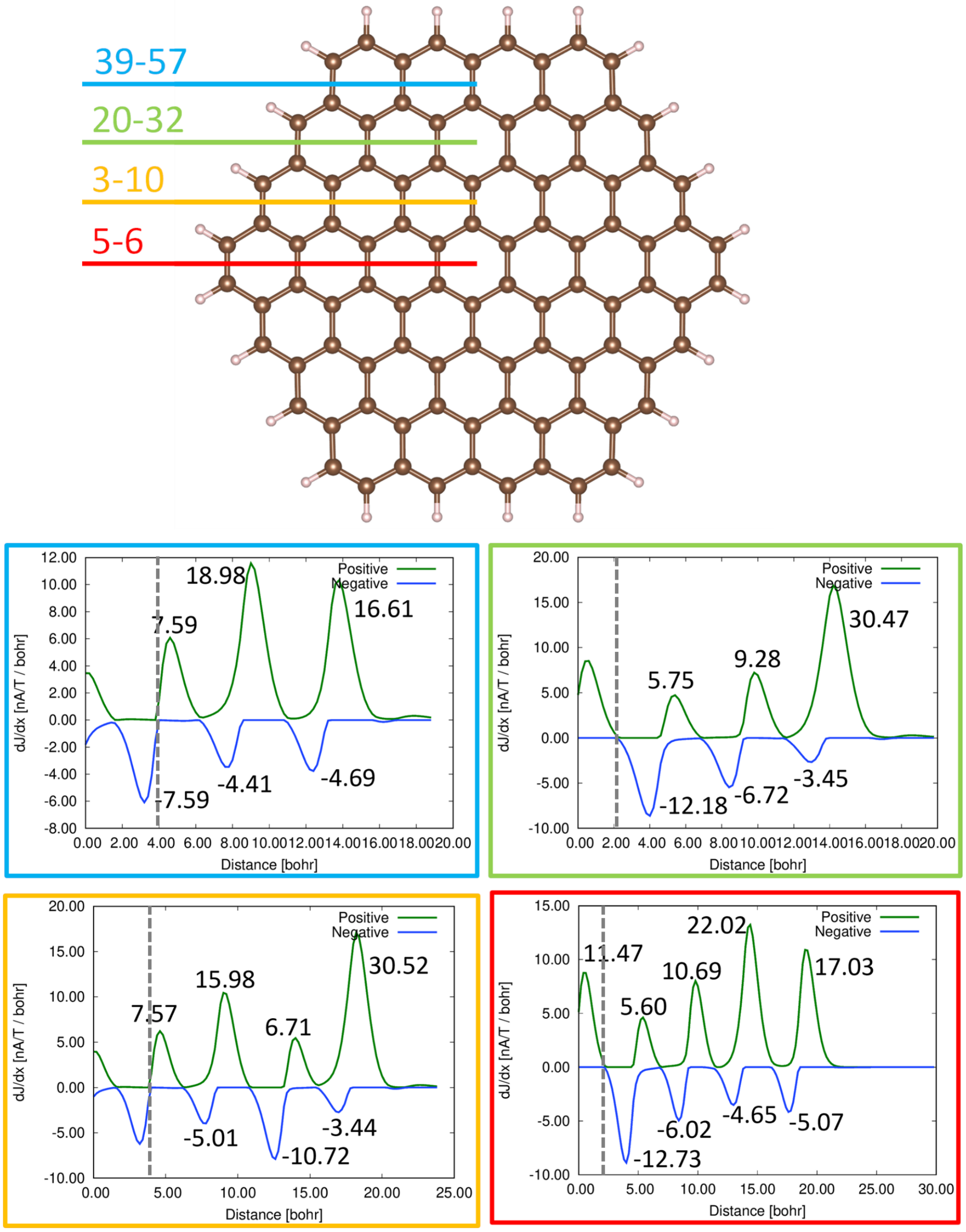

Figure S5: The integration plane and the MIRC profile of circum[2]coronene (AE4). The integration begins in the middle at dashed line. The molecular structures have been made with Vesta[1] and the profile pictures with Gnuplot.[2]

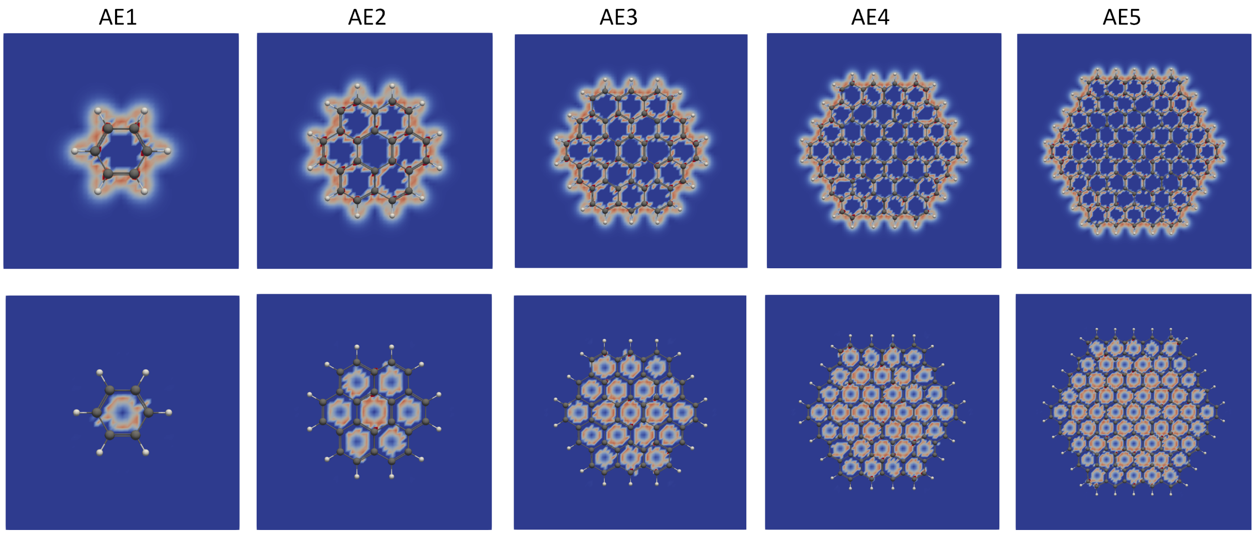

Figure S6: The diatropic (above) and paratropic (below) contributions to the MICD of AE1-AE5 calculated at the CAM-B3LYP/def2-TZVP level.[3,4] The pictures have been made with Paraview.[5]

The MICD of the PP models

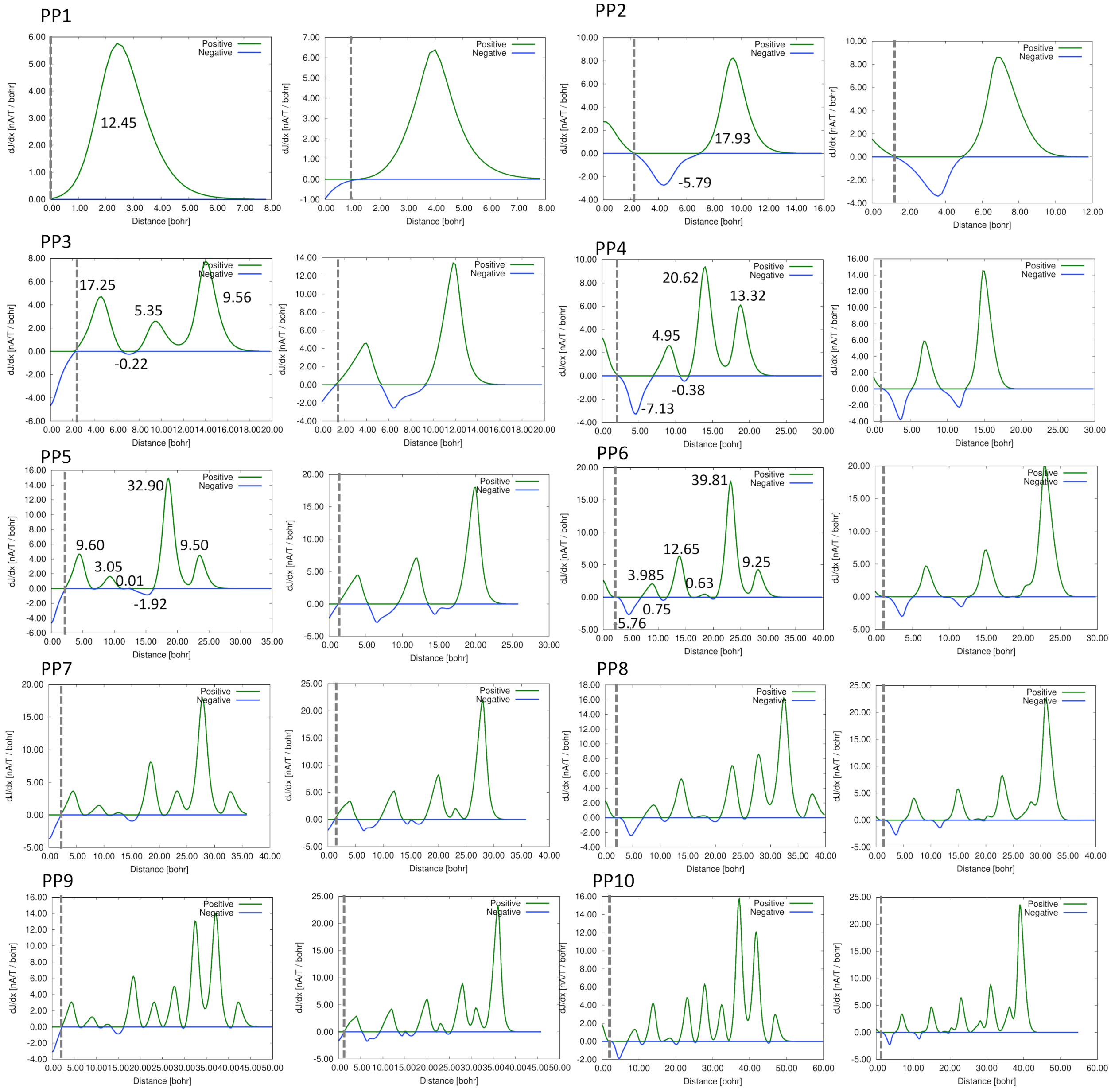

Figure S7: The MIRC profiles of the PPn ($n = 1 - 10$) models calculated at the CAM-B3LYP/def2-TZVP level.[3,4] The picture to the left is along the C_2 axis of the D_{6h} point group. The one to the right is along the σ_d plane of the D_{6h} point group. The profile pictures have been made with Gnuplot.[2]

Table S1: $NICS_{zz}$ values (in ppm) calculated in the center of PPn models at the Hartree-Fock (HF), CAM-B3LYP and ωB97X-D levels using the def2-TZVP basis set for PP1-PP10 and def2-SVP for PP11-PP20.[3,4,6,7]

System	HF	CAM-B3LYP	ωB97X-D
1	11.95	12.35	12.37
2	11.99	13.32	12.76
3	32.67	33.16	32.34
4	34.21	35.58	33.50
5	57.42	56.65	54.88
6	59.28	59.32	56.30
7	83.32	79.71	77.99
8	86.49	82.87	80.43
9	110.72	101.30	101.80
10	116.32	104.11	105.22
11	139.04	112.95	*223.44*
12	149.75	135.04	135.37
13	140.94	131.41	118.91
14	124.62	107.70	113.53
15	157.59	135.02	143.13
16	167.18	139.65	147.91
17	*82.42*	164.72	174.71
18	122.75	130.81	179.24
19	168.88	118.61	162.63
20	188.52	157.66	171.30

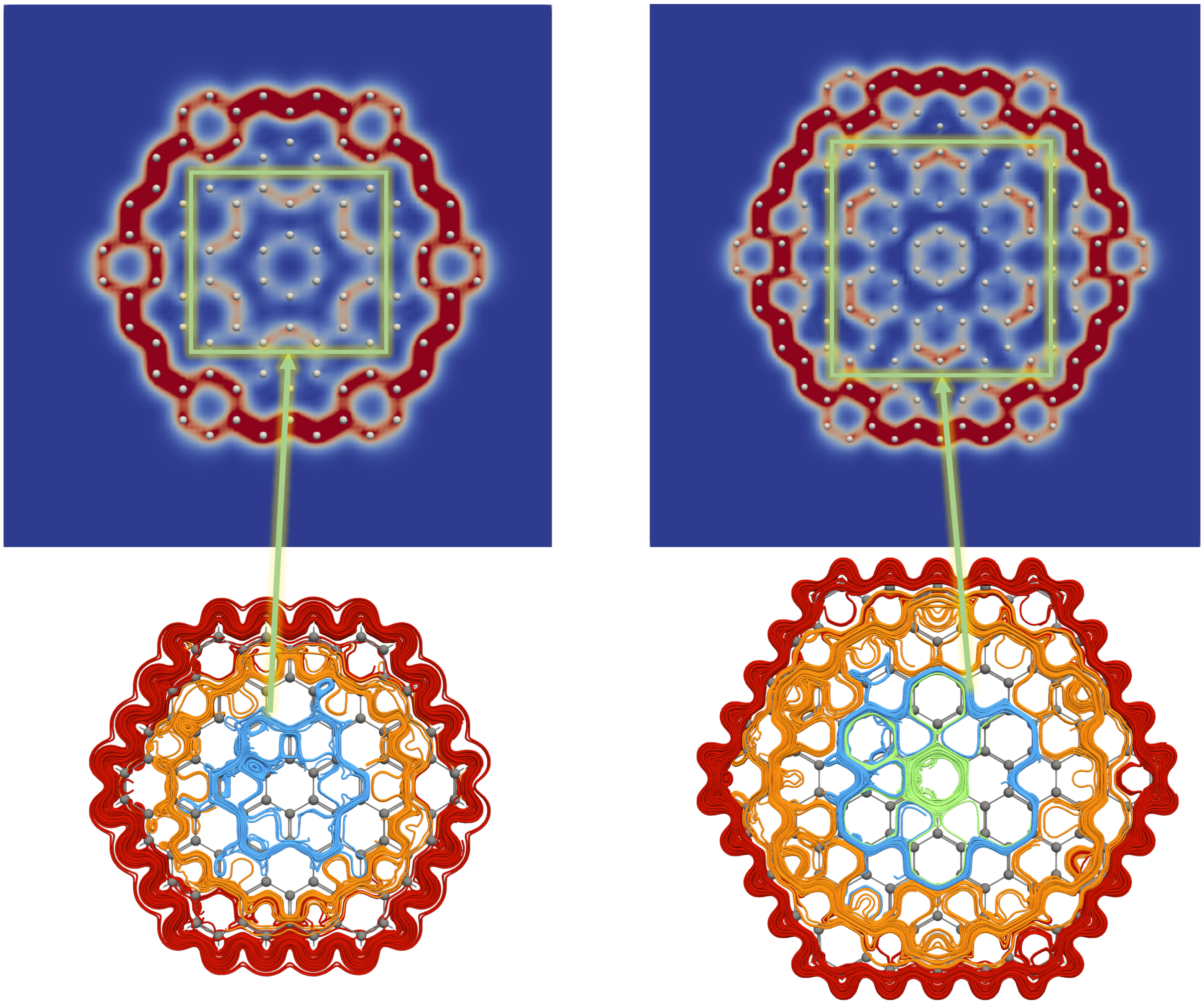

Figure S8: The MICDs of PP4 and PP5 (upper) are compared to the ones for AE4 and AE5 (below). The pictures have been made with Paraview.[5]

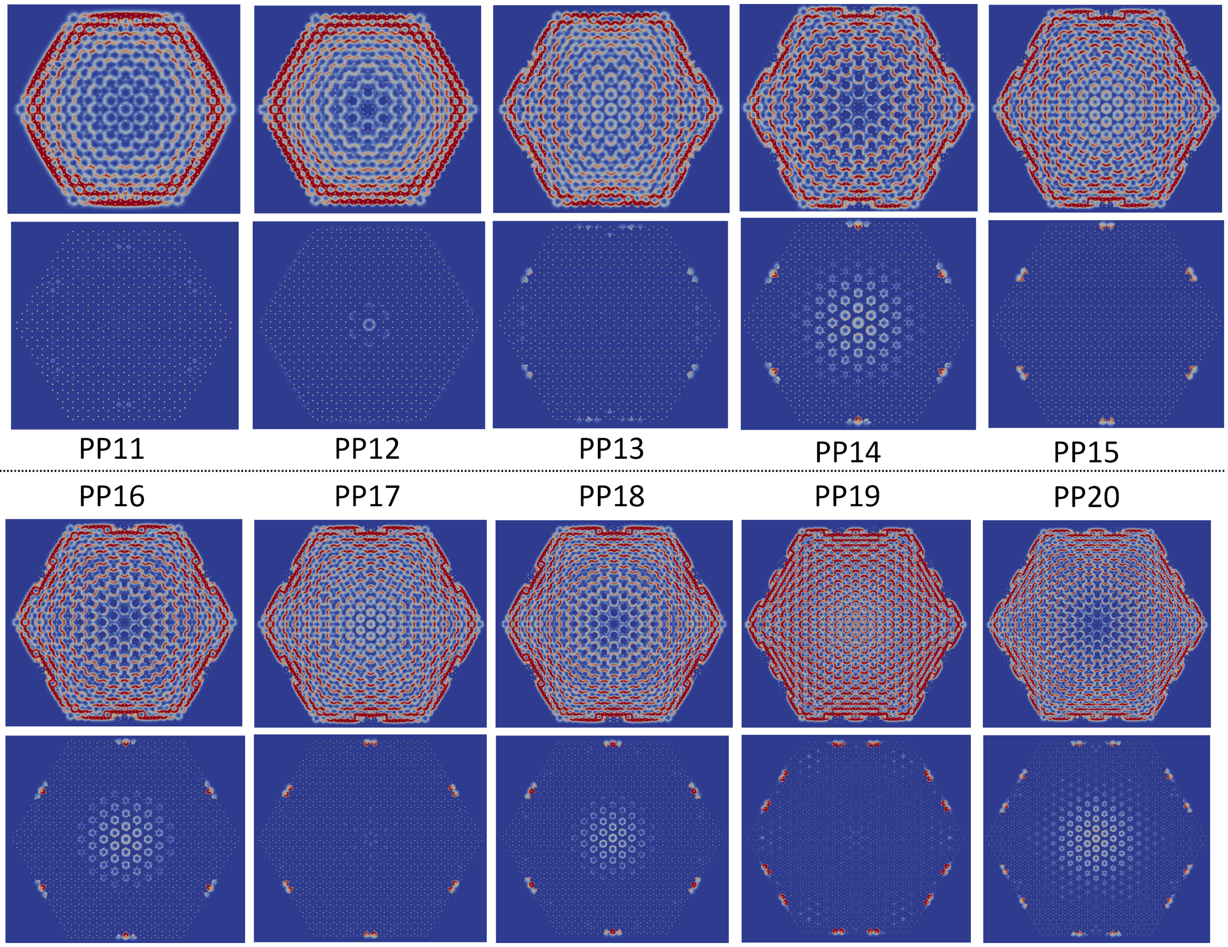

Figure S9: The diatropic (above) and paratropic (below) contributions to the MICD of P11-PP20 calculated at the ωB97X-D/def2-SVP level.[3,4] The pictures have been made with Paraview.[5]

Cartesian coordinates of the molecular structures

Benzene

```
12

C     1.3932572    0.0000002    0.0000000
C     0.6966277    1.2065986    0.0000000
C    -0.6966288    1.2065994    0.0000000
C    -1.3932569    0.0000007    0.0000000
C    -0.6966286   -1.2065984    0.0000000
C     0.6966292   -1.2066000    0.0000000
H     2.4856112    0.0000008    0.0000000
H     1.2428186    2.1525971    0.0000000
H    -1.2428201    2.1525981    0.0000000
H    -2.4856102    0.0000009    0.0000000
H    -1.2428206   -2.1525974    0.0000000
H     1.2428214   -2.1526000    0.0000000
```

Coronene

```
36

C     1.2360904    0.7136555    0.0000000
C     1.2360904   -0.7136555    0.0000000
C     0.0000000    1.4273168    0.0000000
C     2.4593976    1.4199364    0.0000000
C     0.0000000   -1.4273168    0.0000000
C    -1.2360904    0.7136555    0.0000000
C     2.4593976   -1.4199364    0.0000000
C     0.0000000    2.8398686    0.0000000
C     3.6782029    0.6832707    0.0000000
C     2.4308335    2.8437851    0.0000000
C     3.6782029   -0.6832707    0.0000000
C    -1.2360904   -0.7136555    0.0000000
C     1.2473730    3.5270544    0.0000000
C     0.0000000   -2.8398686    0.0000000
C    -2.4593976    1.4199364    0.0000000
C     2.4308335   -2.8437851    0.0000000
C    -1.2473730    3.5270544    0.0000000
C    -2.4593976   -1.4199364    0.0000000
C     1.2473730   -3.5270544    0.0000000
C    -2.4308335    2.8437851    0.0000000
C    -1.2473730   -3.5270544    0.0000000
C    -3.6782029    0.6832707    0.0000000
C    -2.4308335   -2.8437851    0.0000000
C    -3.6782029   -0.6832707    0.0000000
H     4.6220633    1.2335050    0.0000000
```

```
H     3.3792844    3.3860675    0.0000000
H     4.6220633   -1.2335050    0.0000000
H     1.2427801    4.6195788    0.0000000
H     3.3792844   -3.3860675    0.0000000
H    -1.2427801    4.6195788    0.0000000
H     1.2427801   -4.6195788    0.0000000
H    -3.3792844    3.3860675    0.0000000
H    -1.2427801   -4.6195788    0.0000000
H    -4.6220633    1.2335050    0.0000000
H    -3.3792844   -3.3860675    0.0000000
H    -4.6220633   -1.2335050    0.0000000
```

Circumcoronene

```
72
Energy =
C    -1.2248419    0.7071636    0.0000000
C    -1.2248419   -0.7071636    0.0000000
C     0.0000000    1.4143270    0.0000000
C     0.0000000   -1.4143270    0.0000000
C     1.2248419    0.7071636    0.0000000
C     1.2248419   -0.7071636    0.0000000
C    -2.4640721   -1.4226329    0.0000000
C    -2.4640721    1.4226329    0.0000000
C    -0.0000000   -2.8452643    0.0000000
C    -0.0000000    2.8452643    0.0000000
C     2.4640721   -1.4226329    0.0000000
C     2.4640721    1.4226329    0.0000000
C    -2.4630583   -2.8376575    0.0000000
C    -2.4630583    2.8376575    0.0000000
C    -3.6890132   -0.7142427    0.0000000
C    -3.6890132    0.7142427    0.0000000
C     1.2259539   -3.5518985    0.0000000
C     1.2259539    3.5518985    0.0000000
C    -1.2259539   -3.5518985    0.0000000
C    -1.2259539    3.5518985    0.0000000
C     3.6890132   -0.7142427    0.0000000
C     3.6890132    0.7142427    0.0000000
C     2.4630583   -2.8376575    0.0000000
C     2.4630583    2.8376575    0.0000000
C    -3.6980766    3.5451819    0.0000000
C    -3.6980766   -3.5451819    0.0000000
C    -4.9192544    1.4300426    0.0000000
C    -4.9192544   -1.4300426    0.0000000
C     1.2211748   -4.9752165    0.0000000
C     1.2211748    4.9752165    0.0000000
C    -1.2211748    4.9752165    0.0000000
C    -1.2211748   -4.9752165    0.0000000
C     4.9192544   -1.4300426    0.0000000
```

```
C     4.9192544    1.4300426    0.0000000
C     3.6980766   -3.5451819    0.0000000
C     3.6980766    3.5451819    0.0000000
C    -4.8972642    2.8274410    0.0000000
C    -4.8972642   -2.8274410    0.0000000
C    -0.0000000    5.6548735    0.0000000
C    -0.0000000   -5.6548735    0.0000000
C     4.8972642    2.8274410    0.0000000
C     4.8972642   -2.8274410    0.0000000
C    -3.6608710   -4.9847583    0.0000000
C    -3.6608710    4.9847583    0.0000000
C    -6.1473615   -0.6780319    0.0000000
C    -6.1473615    0.6780319    0.0000000
C     2.4864843   -5.6627868    0.0000000
C     2.4864843    5.6627868    0.0000000
C    -2.4864843   -5.6627868    0.0000000
C    -2.4864843    5.6627868    0.0000000
C     6.1473615   -0.6780319    0.0000000
C     6.1473615    0.6780319    0.0000000
C     3.6608710   -4.9847583    0.0000000
C     3.6608710    4.9847583    0.0000000
H    -5.8438409    3.3739506    0.0000000
H    -5.8438409   -3.3739506    0.0000000
H    -0.0000000    6.7478867    0.0000000
H    -0.0000000   -6.7478867    0.0000000
H     5.8438409    3.3739506    0.0000000
H     5.8438409   -3.3739506    0.0000000
H    -4.6097375   -5.5261769    0.0000000
H    -4.6097375    5.5261769    0.0000000
H    -7.0906771   -1.2290659    0.0000000
H    -7.0906771    1.2290659    0.0000000
H     2.4809269    6.7552384    0.0000000
H     2.4809269   -6.7552384    0.0000000
H    -2.4809269   -6.7552384    0.0000000
H    -2.4809269    6.7552384    0.0000000
H     7.0906771    1.2290659    0.0000000
H     7.0906771   -1.2290659    0.0000000
H     4.6097375    5.5261769    0.0000000
H     4.6097375   -5.5261769    0.0000000
```

Circumcircumcoronene

```
120
Energy =
C    -1.2318355    0.7111972    0.0000000
C    -1.2318355   -0.7111972    0.0000000
C     0.0000000    1.4223859    0.0000000
C     0.0000000   -1.4223859    0.0000000
C     1.2318355    0.7111972    0.0000000
```

C	1.2318355	-0.7111972	0.0000000
C	2.4551134	-1.4174551	0.0000000
C	2.4551134	1.4174551	0.0000000
C	0.0000000	-2.8349068	0.0000000
C	0.0000000	2.8349068	0.0000000
C	-2.4551134	-1.4174551	0.0000000
C	-2.4551134	1.4174551	0.0000000
C	3.6867254	-0.7048737	0.0000000
C	3.6867254	0.7048737	0.0000000
C	2.4538036	-2.8403550	0.0000000
C	2.4538036	2.8403550	0.0000000
C	1.2329224	-3.5452315	0.0000000
C	1.2329224	3.5452315	0.0000000
C	-1.2329224	-3.5452315	0.0000000
C	-1.2329224	3.5452315	0.0000000
C	-2.4538036	-2.8403550	0.0000000
C	-2.4538036	2.8403550	0.0000000
C	-3.6867254	-0.7048737	0.0000000
C	-3.6867254	0.7048737	0.0000000
C	4.9225451	-1.4190945	0.0000000
C	4.9225451	1.4190945	0.0000000
C	3.6902480	-3.5535013	0.0000000
C	3.6902480	3.5535013	0.0000000
C	1.2322969	-4.9725963	0.0000000
C	1.2322969	4.9725963	0.0000000
C	-1.2322969	-4.9725963	0.0000000
C	-1.2322969	4.9725963	0.0000000
C	-3.6902480	-3.5535013	0.0000000
C	-3.6902480	3.5535013	0.0000000
C	-4.9225451	-1.4190945	0.0000000
C	-4.9225451	1.4190945	0.0000000
C	-4.9202488	2.8407039	0.0000000
C	-4.9202488	-2.8407039	0.0000000
C	0.0000000	5.6814191	0.0000000
C	0.0000000	-5.6814191	0.0000000
C	4.9202488	2.8407039	0.0000000
C	4.9202488	-2.8407039	0.0000000
C	-6.1446670	0.7110592	0.0000000
C	-6.1446670	-0.7110592	0.0000000
C	-3.6881276	4.9659090	0.0000000
C	-3.6881276	-4.9659090	0.0000000
C	-2.4565386	5.6769648	0.0000000
C	-2.4565386	-5.6769648	0.0000000
C	2.4565386	5.6769648	0.0000000
C	2.4565386	-5.6769648	0.0000000
C	3.6881276	4.9659090	0.0000000
C	3.6881276	-4.9659090	0.0000000
C	6.1446670	0.7110592	0.0000000
C	6.1446670	-0.7110592	0.0000000

C	-6.1534978	3.5527257	0.0000000
C	-6.1534978	-3.5527257	0.0000000
C	0.0000000	7.1054526	0.0000000
C	0.0000000	-7.1054526	0.0000000
C	6.1534978	3.5527257	0.0000000
C	6.1534978	-3.5527257	0.0000000
C	7.3826444	-1.4359089	0.0000000
C	7.3826444	1.4359089	0.0000000
C	4.9348537	-5.6756085	0.0000000
C	4.9348537	5.6756085	0.0000000
C	2.4477888	-7.1115107	0.0000000
C	2.4477888	7.1115107	0.0000000
C	-2.4477888	-7.1115107	0.0000000
C	-2.4477888	7.1115107	0.0000000
C	-4.9348537	-5.6756085	0.0000000
C	-4.9348537	5.6756085	0.0000000
C	-7.3826444	-1.4359089	0.0000000
C	-7.3826444	1.4359089	0.0000000
C	7.3647608	-2.8185937	0.0000000
C	7.3647608	2.8185937	0.0000000
C	6.1233498	-4.9687768	0.0000000
C	6.1233498	4.9687768	0.0000000
C	1.2414096	-7.7873637	0.0000000
C	1.2414096	7.7873637	0.0000000
C	-1.2414096	-7.7873637	0.0000000
C	-1.2414096	7.7873637	0.0000000
C	-6.1233498	-4.9687768	0.0000000
C	-6.1233498	4.9687768	0.0000000
C	-7.3647608	-2.8185937	0.0000000
C	-7.3647608	2.8185937	0.0000000
C	8.6145420	-0.6756644	0.0000000
C	8.6145420	0.6756644	0.0000000
C	4.8924091	-7.1225846	0.0000000
C	4.8924091	7.1225846	0.0000000
C	3.7221255	-7.7982448	0.0000000
C	3.7221255	7.7982448	0.0000000
C	-3.7221255	-7.7982448	0.0000000
C	-3.7221255	7.7982448	0.0000000
C	-4.8924091	-7.1225846	0.0000000
C	-4.8924091	7.1225846	0.0000000
C	-8.6145420	-0.6756644	0.0000000
C	-8.6145420	0.6756644	0.0000000
H	8.3108235	-3.3658101	0.0000000
H	8.3108235	3.3658101	0.0000000
H	7.0702845	-5.5144824	0.0000000
H	7.0702845	5.5144824	0.0000000
H	1.2405379	-8.8802860	0.0000000
H	1.2405379	8.8802860	0.0000000
H	-1.2405379	-8.8802860	0.0000000

```
H    -1.2405379    8.8802860    0.0000000
H    -7.0702845   -5.5144824    0.0000000
H    -7.0702845    5.5144824    0.0000000
H    -8.3108235   -3.3658101    0.0000000
H    -8.3108235    3.3658101    0.0000000
H    -9.5576713    1.2270008    0.0000000
H    -9.5576713   -1.2270008    0.0000000
H    -5.8414483    7.6636842    0.0000000
H    -5.8414483   -7.6636842    0.0000000
H    -3.7162138    8.8906865    0.0000000
H    -3.7162138   -8.8906865    0.0000000
H     3.7162138    8.8906865    0.0000000
H     3.7162138   -8.8906865    0.0000000
H     5.8414483    7.6636842    0.0000000
H     5.8414483   -7.6636842    0.0000000
H     9.5576713    1.2270008    0.0000000
H     9.5576713   -1.2270008    0.0000000
```

Circumcircumcircumcoronene

```
180
Energy =
C     1.2261076    0.7078935    0.0000000
C     1.2261076   -0.7078935    0.0000000
C    -0.0000000   -1.4157871    0.0000000
C    -1.2261076   -0.7078935    0.0000000
C    -1.2261076    0.7078935    0.0000000
C    -0.0000000    1.4157871    0.0000000
C     1.2270034    7.8084979    0.0000000
C     0.0000000    7.1029599    0.0000000
C    -1.2270034   -7.8084979    0.0000000
C     1.2248629    9.2381344    0.0000000
C    -0.0000000    9.9173110    0.0000000
C    -1.2248629   -9.2381344    0.0000000
C     3.6897226    7.8034329    0.0000000
C     2.4614927    7.0990864    0.0000000
C    -3.6897226   -7.8034329    0.0000000
C    -2.4614927   -7.0990864    0.0000000
C     3.6774052    9.2461606    0.0000000
C     2.4794203    9.9197780    0.0000000
C    -3.6774052   -9.2461606    0.0000000
C    -2.4794203   -9.9197780    0.0000000
C     6.1687074    7.8078066    0.0000000
C     4.9131099    7.0971099    0.0000000
C    -6.1687074   -7.8078066    0.0000000
C    -4.9131099   -7.0971099    0.0000000
C     6.1239575    9.2578610    0.0000000
C     4.9555641    9.9324333    0.0000000
C    -6.1239575   -9.2578610    0.0000000
```

C	-4.9555641	-9.9324333	0.0000000
C	7.3510696	7.1071300	0.0000000
C	-7.3510696	7.1071300	0.0000000
C	1.2264246	3.5482679	0.0000000
C	0.0000000	2.8403779	0.0000000
C	-1.2264246	-3.5482679	0.0000000
C	1.2257983	4.9654856	0.0000000
C	0.0000000	5.6722214	0.0000000
C	-1.2257983	-4.9654856	0.0000000
C	3.6873376	3.5443153	0.0000000
C	2.4596779	2.8362488	0.0000000
C	-3.6873376	-3.5443153	0.0000000
C	-2.4596779	-2.8362488	0.0000000
C	3.6849722	4.9708537	0.0000000
C	2.4623995	5.6767064	0.0000000
C	-3.6849722	-4.9708537	0.0000000
C	-2.4623995	-5.6767064	0.0000000
C	6.1513437	3.5514799	0.0000000
C	4.9122878	2.8361107	0.0000000
C	-6.1513437	-3.5514799	0.0000000
C	-4.9122878	-2.8361107	0.0000000
C	6.1488559	4.9668650	0.0000000
C	4.9172428	5.6812584	0.0000000
C	-6.1488559	-4.9668650	0.0000000
C	-4.9172428	-5.6812584	0.0000000
C	8.6128905	3.5583048	0.0000000
C	7.3758593	2.8416329	0.0000000
C	-8.6128905	-3.5583048	0.0000000
C	-7.3758593	-2.8416329	0.0000000
C	8.5886433	4.9586555	0.0000000
C	7.3880276	5.6798296	0.0000000
C	-8.5886433	-4.9586555	0.0000000
C	-7.3880276	-5.6798296	0.0000000
C	9.8304899	2.8126480	0.0000000
C	-9.8304899	2.8126480	0.0000000
C	3.6861025	-0.7120191	0.0000000
C	2.4598394	-1.4201890	0.0000000
C	-3.6861025	0.7120191	0.0000000
C	-2.4598394	1.4201890	0.0000000
C	3.6861025	0.7120191	0.0000000
C	2.4598394	1.4201890	0.0000000
C	-3.6861025	-0.7120191	0.0000000
C	-2.4598394	-1.4201890	0.0000000
C	6.1473717	-0.7058527	0.0000000
C	4.9131359	-1.4211703	0.0000000
C	-6.1473717	0.7058527	0.0000000
C	-4.9131359	1.4211703	0.0000000
C	6.1473717	0.7058527	0.0000000
C	4.9131359	1.4211703	0.0000000

C	-6.1473717	-0.7058527	0.0000000
C	-4.9131359	-1.4211703	0.0000000
C	8.6028324	-0.7063230	0.0000000
C	7.3787355	-1.4178280	0.0000000
C	-8.6028324	0.7063230	0.0000000
C	-7.3787355	1.4178280	0.0000000
C	8.6028324	0.7063230	0.0000000
C	7.3787355	1.4178280	0.0000000
C	-8.6028324	-0.7063230	0.0000000
C	-7.3787355	-1.4178280	0.0000000
C	11.0795216	-0.6745722	0.0000000
C	9.8461126	-1.4383540	0.0000000
C	-11.0795216	0.6745722	0.0000000
C	-9.8461126	1.4383540	0.0000000
C	11.0795216	0.6745722	0.0000000
C	9.8461126	1.4383540	0.0000000
C	-11.0795216	-0.6745722	0.0000000
C	-9.8461126	-1.4383540	0.0000000
C	1.2257983	-4.9654856	0.0000000
C	0.0000000	-5.6722214	0.0000000
C	-1.2257983	4.9654856	0.0000000
C	1.2264246	-3.5482679	0.0000000
C	0.0000000	-2.8403779	0.0000000
C	-1.2264246	3.5482679	0.0000000
C	3.6849722	-4.9708537	0.0000000
C	2.4623995	-5.6767064	0.0000000
C	-3.6849722	4.9708537	0.0000000
C	-2.4623995	5.6767064	0.0000000
C	3.6873376	-3.5443153	0.0000000
C	2.4596779	-2.8362488	0.0000000
C	-3.6873376	3.5443153	0.0000000
C	-2.4596779	2.8362488	0.0000000
C	6.1488559	-4.9668650	0.0000000
C	4.9172428	-5.6812584	0.0000000
C	-6.1488559	4.9668650	0.0000000
C	-4.9172428	5.6812584	0.0000000
C	6.1513437	-3.5514799	0.0000000
C	4.9122878	-2.8361107	0.0000000
C	-6.1513437	3.5514799	0.0000000
C	-4.9122878	2.8361107	0.0000000
C	8.5886433	-4.9586555	0.0000000
C	7.3880276	-5.6798296	0.0000000
C	-8.5886433	4.9586555	0.0000000
C	-7.3880276	5.6798296	0.0000000
C	8.6128905	-3.5583048	0.0000000
C	7.3758593	-2.8416329	0.0000000
C	-8.6128905	3.5583048	0.0000000
C	-7.3758593	2.8416329	0.0000000
C	9.8304899	-2.8126480	0.0000000

C	-9.8304899	-2.8126480	0.0000000
C	1.2248629	-9.2381344	0.0000000
C	0.0000000	-9.9173110	0.0000000
C	-1.2248629	9.2381344	0.0000000
C	1.2270034	-7.8084979	0.0000000
C	-0.0000000	-7.1029599	0.0000000
C	-1.2270034	7.8084979	0.0000000
C	3.6774052	-9.2461606	0.0000000
C	2.4794203	-9.9197780	0.0000000
C	-3.6774052	9.2461606	0.0000000
C	-2.4794203	9.9197780	0.0000000
C	3.6897226	-7.8034329	0.0000000
C	2.4614927	-7.0990864	0.0000000
C	-3.6897226	7.8034329	0.0000000
C	-2.4614927	7.0990864	0.0000000
C	6.1239575	-9.2578610	0.0000000
C	4.9555641	-9.9324333	0.0000000
C	-6.1239575	9.2578610	0.0000000
C	-4.9555641	9.9324333	0.0000000
C	6.1687074	-7.8078066	0.0000000
C	4.9131099	-7.0971099	0.0000000
C	-6.1687074	7.8078066	0.0000000
C	-4.9131099	7.0971099	0.0000000
C	7.3510696	-7.1071300	0.0000000
C	-7.3510696	-7.1071300	0.0000000
H	12.0223286	-1.2263256	0.0000000
H	-12.0223286	1.2263256	0.0000000
H	9.5349468	-5.5050041	0.0000000
H	-9.5349468	5.5050041	0.0000000
H	10.7764401	-3.3599850	0.0000000
H	-10.7764401	-3.3599850	0.0000000
H	7.0731934	-9.7984792	0.0000000
H	-7.0731934	9.7984792	0.0000000
H	8.2980524	-7.6526784	0.0000000
H	-8.2980524	-7.6526784	0.0000000
H	7.0731934	9.7984792	0.0000000
H	-7.0731934	-9.7984792	0.0000000
H	8.2980524	7.6526784	0.0000000
H	-8.2980524	7.6526784	0.0000000
H	9.5349468	5.5050041	0.0000000
H	-9.5349468	-5.5050041	0.0000000
H	10.7764401	3.3599850	0.0000000
H	-10.7764401	3.3599850	0.0000000
H	12.0223286	1.2263256	0.0000000
H	-12.0223286	-1.2263256	0.0000000
H	0.0000000	-11.0100082	0.0000000
H	2.4783876	-11.0126634	0.0000000
H	-2.4783876	11.0126634	0.0000000
H	4.9491352	-11.0248048	0.0000000

H	-4.9491352	11.0248048	0.0000000
H	0.0000000	11.0100082	0.0000000
H	2.4783876	11.0126634	0.0000000
H	-2.4783876	-11.0126634	0.0000000
H	4.9491352	11.0248048	0.0000000
H	-4.9491352	-11.0248048	0.0000000

References

(1) Momma, K.; Izumi, F. VESTA 3 for three-dimensional visualization of crystal, volumetric and morphology data. *J. Appl. Crystallogr.* **2011**, *44*, 1272–1276.

(2) Williams, T.; Kelley, C.; many others, Gnuplot 5.4: an interactive plotting program. http://gnuplot.sourceforge.net/ (Last accessed: October 2024).

(3) Yanai, T.; Tew, D. P.; Handy, N. C. A new hybrid exchange–correlation functional using the Coulomb-attenuating method (CAM-B3LYP). *Chem. Phys. Lett.* **2004**, *393*, 51–57.

(4) Weigend, F.; Ahlrichs, R. Balanced basis sets of split valence, triple zeta valence and quadruple zeta valence quality for H to Rn: Design and assessment of accuracy. *Phys. Chem. Chem. Phys.* **2005**, *7*, 3297–3305.

(5) Ahrens, J.; Geveci, B.; Law, C. ParaView: An End-User Tool for Large Data Visualization, Visualization Handbook, Elsevier, 2005, ISBN-13: 978-0123875822, see also: http://www.paraview.org.

(6) Chai, J.-D.; Head-Gordon, M. Long-range corrected hybrid density functionals with damped atom–atom dispersion corrections. *Phys. Chem. Chem. Phys.* **2008**, *10*, 6615–6620.

(7) Schäfer, A.; Horn, H.; Ahlrichs, R. Fully optimized contracted Gaussian basis sets for atoms Li to Kr. *J. Chem. Phys.* **1992**, *97*, 2571–2577.
